# Meadowsweet Teas as New Functional Beverages: Comparative Analysis of Nutrients, Phytochemicals and Biological Effects of Four *Filipendula* Species

**DOI:** 10.3390/molecules22010016

**Published:** 2016-12-26

**Authors:** Daniil N. Olennikov, Nina I. Kashchenko, Nadezhda K. Chirikova

**Affiliations:** 1 Institute of General and Experimental Biology, Siberian Division, Russian Academy of Science, Sakh’yanovoy Street, 6, Ulan-Ude 670047, Russia; ninkk@mail.ru; 2 Department of Biochemistry and Biotechnology, North-Eastern Federal University, 58 Belinsky Street, Yakutsk 677027, Russia; hofnung@mail.ru

**Keywords:** *Filipendula camschatica*, *Filipendula denudata*, *Filipendula stepposa*, *Filipendula ulmaria*, tannins, flavonoids, polysaccharides, essential oils, anti-diabetic activity, antioxidant activity, anti-complement activity

## Abstract

In recent years, the increased popularity of functional beverages such as herbal teas and decoctions has led to the search for new sources of raw materials that provide appropriate taste and functionality to consumers. The objective of this study was to investigate the nutritional, phytochemical profiles and bioactivities of possible functional beverages produced from *F. ulmaria* and its alternative substitutes (*F. camtschatica*, *F. denudata*, *F. stepposa*). The investigated decoctions were analyzed regarding their macronutrient, carbohydrate, organic acid, amino acid and mineral composition. Quantification of the main phenolic compounds in the decoctions of meadowsweet floral teas was performed by a microcolumn RP-HPLC-UV procedure; the highest content was revealed in *F. stepposa* tea. The investigation of the essential oil of four meadowsweet teas revealed the presence of 28 compounds, including simple phenols, monoterpenes, sesquiterpenes and aliphatic components. The dominance of methyl salicylate and salicylaldehyde was noted in all samples. Studies on the water soluble polysaccharides of *Filipendula* flowers allowed us to establish their general affiliation to galactans and/or arabinogalactans with an admixture of glucans of the starch type and galacturonans as minor components. The bioactivity data demonstrated a good ability of meadowsweet teas to inhibit amylase, α-glucosidase and AGE formation. Tea samples showed antioxidant properties by the DPPH^•^, ABTS^•+^ and Br^•^ free radicals scavenging assays and the carotene bleaching assay, caused by the presence of highly active ellagitannins. The anti-complement activity of the water-soluble polysaccharide fraction of meadowsweet teas indicated their possible immune-modulating properties. *Filipendula* beverage formulations can be expected to deliver beneficial effects due to their unique nutritional and phytochemical profiles. Potential applications as health-promoting functional products may be suggested.

## 1. Introduction

The food and beverage industry is growing solidly due to the increased demand of consumers. The business opportunity has attracted the herbal industry to penetrate the herbal-based food product market, and herbal-based products are becoming a widespread production trend among manufacturers [1]. From a global perspective and particularly with regards to products sold with a specific medical claim, Europe and Asia have been leading the way in terms of supplying herbal medicinal products [2]. Herbal teas are one type of herbal medicinal product. The term generally refers to any functional beverage made from an infusion or decoction of herbs, spices, or other plant materials in hot water. These beverages usually do not contain caffeine. The consumption of functional beverages has grown in popularity, primarily due to metabolism, immunity and oxidant status improving claims [3,4,5,6]. 

One of the most popular plant genera for herbal tea preparation is *Filipendula* [7]. This genus includes perennial herbaceous plants of the family Rosaceae that number about 30 species, commonly known as meadowsweets. These herbs are used due to the specific honey-like fragrance of the flowers and the pleasant taste of water decoctions [8]. Scientifically, the most studied species are *F. ulmaria* (L.) Maxim. and *F. vulgaris* Moench, which are officinal plant species in many countries. Researchers have found in these species derivatives of salicylic acid [9,10], 2-pyrone-4,6-dicarboxylic acid [11,12], flavonoids [13,14], tannins [15,16] and essential oils [17,18]. 

The growing demand for use of an officinal species, coupled with the simultaneous shortage of raw materials due to its low adaptability, agricultural peculiarities and unlimited gathering as a medicinal plant, necessitates scouting studies for the discovery of vicarious and chemically similar substitutes within the genus *Filipendula* [19,20]. Species *F. denudata* and *F. stepposa* relate to the same Ulmaria subgenus as *F. ulmaria* and species *F. camtschatica* belongs to the close subgenus Aceraria; accordingly, these plant species may be potential substitutes for the officinal species *F. ulmaria*. Until recently, the chemical composition of these species was not known. The presence of several compounds has been previously demonstrated for the genus *Filipendula*, such as dimeric and monomeric ellagitannins, in addition to flavonol-4′-*O*-glycosides [21]. Moreover, it is known that these plant species have a positive effect in diabetic diseases, illnesses of the immune system and may have possible antioxidant effects [22,23,24]. None of these aspects has been previously studied for the species *F. camschatica*, *F. stepposa* and *F. denudata*.

In this regard, the objective of this study was to comparatively investigate the nutritional composition (macronutrients, carbohydrates, organic acids, amino acids and minerals), and phytochemical profiles (phenolics, essential oils, polysaccharides) and biological effects of four meadowsweet teas (*F. camschatica*, *F. denudata*, *F. stepposa* and *F. ulmaria*).

## 2. Results and Discussion

### 2.1. Nutritional Profiles of Meadowsweet Teas

The results of the nutritional characterization of the meadowsweet floral teas, including organoleptic parameters (color, odor and taste) and the macronutrient composition are presented in Table 1. Teas extractives ranged from 310 mg/100 mL (*F. camschatica*) to 394 mg/100 mL (*F. stepposa*), demonstrating the good extractibility of *Filipendula* tea components in boiling water. The color of the four meadowsweet tea was typical for yellow floral teas, the odor was characterized as specific, methyl salicylate-like, and the taste was characterized as astringent, with bitterness of variable intensity. In general, these organoleptic characteristics allow us to describe these meadowsweet floral teas as specific products with atypical but pleasant tastes and odours. Concerning the macronutrients, carbohydrates were the most abundant compounds, reaching 132.05–173.81 mg/100 mL decoction, followed by proteins (8.57–12.49 mg/100 mL decoction). Moderate ash amount parameters (34.40–54.59 mg/100 mL decoction) were typical for *Filipendula* floral teas [25,26]. Low lipid (<1 mg/100 mL decoction) and energy (0.57–0.75 kcal/100 mL decoction) contents were detected in the meadowsweet floral teas.

The free sugar compositions of the four samples of meadowsweet teas are shown in Table 1. Glucose was found to be predominant in all samples, accounting for 71%–77% of the total free sugars. This compound was followed by sucrose and galactose, the contents of which were 10.18–15.61 and 8.07–11.22 mg/100 mL, respectively. Only trace amounts of fructose were detected in the meadowsweet teas. The total content of free sugars in the four *Filipendula* decoctions ranged from 74.32 to 93.14 mg/100 mL.

The contents of organic acids detected in the meadowsweet teas were also investigated. Citric and malic acids were present in the examined tea samples at the highest levels, i.e., 5.84–10.84 and 6.52–11.67 mg per 100 mL of decoction, respectively. Their proportions of the total organic acids content were 26%–37% and 30%–44%, respectively. The amount of oxalic acid, a known antinutrient, was minimal in *F. stepposa* tea (4.93 mg/100 mL) and maximal in *F. camschatica* tea (9.62 mg/100 mL). Some other acids like quinic, succinic and tartaric acid were found in trace or low levels.

Nineteen amino acids were identified and quantified in the investigated tea samples. Aspartic acid was predominant in *F. denudata* (0.90 mg/100 mL), *F. stepposa* (3.71 mg/100 mL) and *F. ulmaria* (2.03 mg/100 mL) teas, whereas glutamic acid was the main amino acid in *F. camschatica* tea (3.65 mg/100 mL). The percentages of the basic amino acids (aspartic acid, glutamic acid and leucine) were 25% (*F. ulmaria* tea), 38% (*F. camschatica* tea), 41% (*F. denudata* tea) and 47% (*F. stepposa* tea) of the total amino acid content. The lowest concentrations (<0.1 mg/100 mL) in teas were observed for glutamine in *F. camschatica*; cysteine, glutamine and histidine in *F. denudata*; cysteine and tyrosine in *F. stepposa* and cysteine and glutamine in *F. ulmaria*.

The content of nine microelements (Cr, Co, Cu, Fe, Mn, Mo, Ni, Se and Zn) and two macroelements (Ca and Mg) in the meadowsweet floral teas were determined. Among the major micronutrients measured in these floral teas, Mg predominated, but high levels of Ca were also found. High levels of Fe (11.12 μg/100 mL) and Mn (6.30 μg/100 mL) were observed in *F. ulmaria* floral tea compared with the other teas. *F. camschatica* floral tea was distinguished by high contents of Mg (4923.48 μg/100 mL), Ca (396.29 μg/100 mL), Se (0.34 μg/100 mL) and Co (0.11 μg/100 mL). The highest amounts of Cu (5.31 μg/100 mL), Mo (0.08 μg/100 mL) and Zn (16.14 μg/100 mL) were observed in *F. denudata* tea, whereas *F. stepposa* accumulated Cr (0.29 μg/100 mL) and Ni (1.43 μg/100 mL). Although the general trends in the concentration of major minerals were similar across the *Filipendula* species, substantial variation in the levels of individual elements occurred. In the absence of information on factors such as soil type and seasonal climate, a more detailed comparison is not justified. In order to estimate whether the floral teas prepared from *Filipendula* species represent an important source of microelements, these results were compared with the recommended daily dietary requirements. The concentrations of the micronutrients dissolved in floral teas are below the levels considered to be adequate to humans [27]. Our results provide evidence that the meadowsweet teas are the products with appropriate nutritional value and good sugars, organic acids, amino acids, macro- and micro-mineral content.

### 2.2. General Phytochemical Characteristics of Meadowsweet Teas

The contents of the major groups of phenolic compounds (flavonoids, tannins, catechins and proanthocyanidins) were determined for the phytochemical characterization of the meadowsweet floral teas. Water soluble (WS) polymers like polysaccharides are an obligate group of phytochemicals in all herbal teas. The potential bioactivity of WS-polysaccharides necessitated us to investigate their contents. The data presented in Table 2 demonstrate the general phytochemical characteristics of meadowsweet teas.

The amount of flavonoids varied from 18.34 mg/100 mL (*F. camschatica*) to 55.31 mg/100 mL (*F. ulmaria*). The highest concentration of tannins was observed in *F. stepposa* tea (120.18 mg/100 mL), whereas the lowest concentration was detected in *F. camschatica* tea (51.83 mg/100 mL). The content of catechins changed from 1.27 mg/100 mL (*F. denudata*) to 9.57 mg/100 mL (*F. camschatica*), while in *F. stepposa*, trace amounts of catechins were observed. The amount of proanthocyanidins varied from 2.15 mg/100 mL (*F. denudata*) to 5.14 mg/100 mL (*F. stepposa*). The difference between WS-polysaccharide contents was not significant, i.e., from 21.39 mg/100 mL (*F. camschatica*) to 30.47 mg/100 mL (*F. stepposa*). The general phytochemical profile results show that meadowsweet floral teas are a good source of phenolic compounds as well as WS-polysaccharides.

### 2.3. MC-RP-HPLC Quantification of the Main Phenolics of Meadowsweet Teas

**Figure 1:**
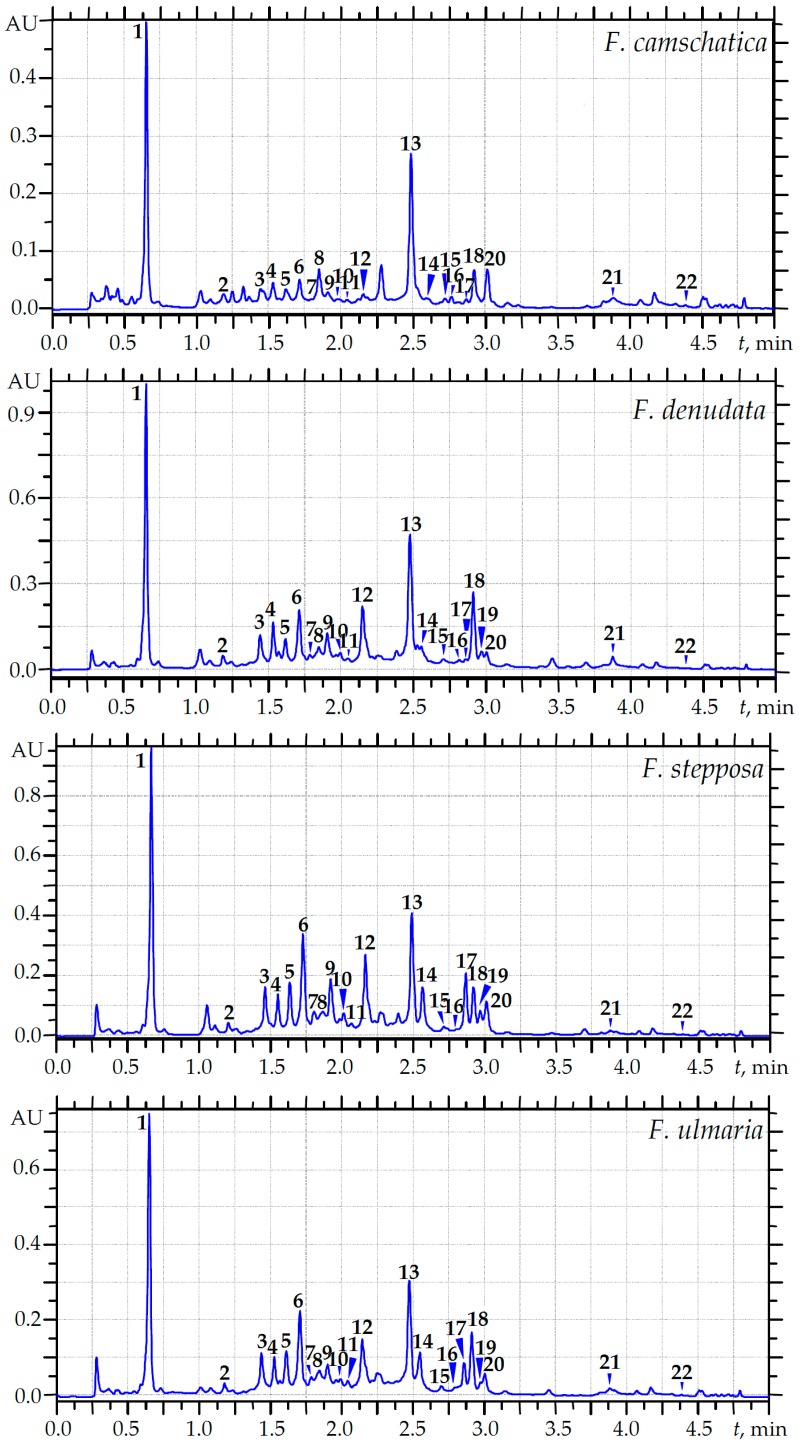
MC-RP-HPLC-UV chromatograms of four meadowsweet floral tea decoctions at 270 nm. Compounds: **1**—gallic acid; **2**—protocatechuic acid; **3**—tellimagrandin I_1_; **4**—rugosin B_1_; **5**—tellimagrandin I_2_; **6**—rugosin B_2_; **7**—caffeic acid; **8**—1,3-di-*O*-caffeoylquinic acid; **9**—rugosin E_1_; **10**—rugosin E_2_; **11**—tellimagrandin II; **12**—rugosin D; **13**—ellagic acid; **14**—quercetin-3-*O*-β-d-glucoside (isoquercitrin); **15**—quercetin-3-*O*-α-l-arabinoside (avicularin); **16**—quercetin-3-*O*-β-d-glucuronide (miquelianin); **17**—quercetin-3-*O*-α-l-rhamnoside (quercitin); **18**—quercetin-4′-*O*-β-d-glucoside (spiraeoside); **19**—kaempferol-3-*O*-α-l-rhamnoside (afzelin); **20**—kaempferol-4′-*O*-β-d-glucoside; **21**—quercetin; **22**—methyl salicylate.

The presence of different classes of compounds, including simple phenolics, phenylpropanoids, catechins, flavonoids and ellagitannins, has been found in the flowers of *F. camschatica*, *F. denudata*, *F. stepposa* and *F. ulmaria* [21]. The present work aimed at the quantification of some principal compounds in meadowsweet floral teas by a microcolumn (MC)-RP-HPLC-UV procedure. Among the known phenolics described in *Filipendula* flowers, only 22 were found in quantifiable amounts in meadowsweet teas, including eight flavonols, eight ellagitannins, protocatechuic, gallic, ellagic, caffeic and 1,3-di-*O*-caffeoylquinic acids, and methyl salicylate (Figure 1). These contents are summarized in Table 3. 

The amounts of the specific compounds varied among the different *Filipendula* species. The results show that the total content of quantifiable components in the meadowsweet tea samples displayed a 3.6-fold variation. *F. stepposa* tea had the highest total compound content (214.25 mg/100 mL), followed in order by *F. denudata* tea (184.09 mg/100 mL), *F. ulmaria* tea (137.62 mg/100 mL) and *F. camschatica* tea (59.07 mg/100 mL). 

As we mentioned in our previous work, dimeric and monomeric ellagitannins are one of the chemotaxonomic markers of the *Filipendula* genus [21]. The compositions of ellagitannins in the investigated floral teas were typical, with a predominance of dimers over monomers. The principal constituents of this class were rugosins. A high content of rugosin D (15.62 mg/100 mL) was observed in the *F. denudata* tea sample and the dominance of rugosin B_2_ was typical for the *F. stepposa*, *F. ulmaria* and *F. camschatica* decoctions (22.44, 12.36 and 2.92 mg/100 mL, respectively). 

Other marker compounds of the genus *Filipendula* are flavonoids, quercetin and kaempferol derivatives, found in the investigated decoctions primarily as the glycoside form in monoglycosides. The 4′-*O*-glycosides made up the principal part of the flavonoids of tea samples, and the predominance of quercetin-4′-*O*-β-D-glucoside (spiraeoside) was observed in all decoctions investigated. The highest content of spiraeoside was revealed in the *F. denudata* decoction (28.50 mg/100 mL) while the lowest was in *F. camschatica* tea (6.43 mg/100 mL). It should be noted that the percentage of kaempferol derivatives was high in *F. camschatica* tea (37.42%), whereas in other tea samples this value did not exceed 18% and the prevalence of quercetin derivatives was higher.

Speculating about the possible reasons for these results, it can be concluded that *F. ulmaria*, *F. denudata* and *F. stepposa* are relate to the same subgenus Ulmaria of the *Filipendula* genus, while *F. camschatica* belong to the subgenus Aceraria. Due to their high content of flavonoids and ellagitannins, *F. denudata* and *F. stepposa* may be considered as promising substitutes for the officinal species *F. ulmaria*.

The highest content of gallic and protocatechuic acids was noted in samples of *F. stepposa* tea (26.17 mg/100 mL and 1.32 mg/100 mL, respectively), while the lowest concentration of gallic acid was found in *F. camschatica* tea (11.80 mg/100 mL) and the lowest content of protocatechuic acid was revealed in *F. ulmaria* tea sample (0.73 mg/100 mL). The content of methyl salicylate varied slightly from 0.73 mg/100 mL (*F. ulmaria* tea) to 0.97 mg/100 mL (*F. stepposa* tea). Quantifiable amounts of phenylpropanoids are presented by caffeic and 1,3-di-*O*-caffeoylquinic acid; the highest content of both compounds was noted in samples of *F. stepposa* tea (7.66 and 12.47 mg/100 mL, respectively), while the lowest was noticed for *F. camschatica* tea (1.08 and 6.18 mg/100 mL, respectively). The *F. denudata* decoction was distinguished by the highest content of ellagic acid (26.72 mg/100 mL), whereas *F. camschatica* tea contained the least amount of this compound (10.92 mg/100 mL). 

The data allowed us to calculate the total uptake of specific compounds after application of the standard dosage of meadowsweet teas (100 mL). This was 59.07 mg/100 mL for *F. camschatica* floral tea, 137.62 mg/100 mL for *F. ulmaria* floral tea, 184.09 mg/100 mL for *F. denudata* floral tea and 214.25 mg/100 mL for *F. stepposa* floral tea. The HPLC quantification results are clear evidence that *Filipendula* species accumulate phenolic compounds and their decoctions are a good source of flavonols, ellagitannins, simple phenols and phenylpropanoids. 

### 2.4. Essential Oil Compositions of Meadowsweet Flowers

All samples of these meadowsweet teas were characterized by specific odours caused by the presence of essential oil components. The most studied essential oil of the *Filipendula* genus is *F. ulmaria* essential oil, which consists of salicylic acid derivatives like salicylaldehyde, methyl salicylate and other components responsible for the characteristic odors of the meadowsweet teas [28]. The other species investigated in the present work (*F. camschatica*, *F. denudata*, *F. stepposa*) have similar odours, but have not yet been assessed regarding their essential oil composition. The samples of the floral essential oils were isolated by hydrodistillation and the compositions were determined after GC/MS analysis. The yields of the essential oil were 0.02%–0.11%. All the samples were yellowish liquids with strong odors. Twenty-eight compounds were identified in the four essential oils, including simple phenols, monoterpenes, sesquiterpenes and aliphatic components (Table 4).

As expected, salicylaldehyde (35.7%) and methyl salicylate (18.4%) were the major components of *F. ulmaria* essential oil, as well as heptadecanal (6.9%) and hexadecanal (5.2%). The contents of salicylaldehyde and methyl salicylate in *F. stepposa* essential oil were 25.7% and 14.2%, respectively. This essential oil was characterized by the highest amount of aliphatic compounds like heptadecanal (9.4%), hexadecanal (6.3%) and decanal (2.9%) as well as the phenol vanillin (5.9%) and monoterpene linalool (5.7%). With respect to *F. denudata* essential oil, the composition was close to that of *F. ulmaria* essential oil, with a high content of salicylaldehyde (45.9%) and a moderate amount of methyl salicylate (20.7%). The flowers of *F. camschatica* produced an essential oil with a high content of methyl salicylate (73.9%), corroborated by the odor, which was described as very sharp. Salicylaldehyde (9.0%), ethyl salicylate (5.2%), benzyl salicylate (4.4%) and hexadecanal (2.9%) were also detected. 

The data on the chemical composition of the essential oils of the *Filipendula* genus indicate the similarity of the volatile compounds. The main components of the essential oil from *F. vulgaris* (*F. hexapetala*) flowering shoots were *n*-tricosane (17.9%), salicylaldehyde (13.7%), benzyl salicylate (6.8%), methyl salicylate (6.7%) [18] and in the essential oil from *F. vulgaris* leaves salicylaldehyde (68.6%), 3-hexen-1-ol (6.0%), α-asarone (5.9%) and 2-hexenal (4.2%) [17]. Methyl salicylate (70.1%) together with eicosane (4.6%) and tricosane (2.3%) dominated in the essential oil from the flowers of *F. palmata* [29]. 

### 2.5. Water-Soluble Polysaccharide Characterization of Meadowsweet Teas

The yields of the WSP fraction from meadowsweet flowers ranged from 13.03 mg/g (*F. denudata*) to 17.24 mg/g (*F. ulmaria*) (Table 5). All WSP had a high carbohydrate content (973.64–989.47 mg/g) and low uronic acid (63.18–102.27 mg/g) and protein amounts (12.60–23.18 mg/g). The negative reaction with resorcinol declared the absence of fructose-containing polymers like inulin. However, positive reactions with iodine and the Yariv reagent indicated the presence of starch-related polysaccharides and arabinogalactan-protein complexes. The monosaccharide compositions of four WSP fractions from *Filipendula* flowers showed a dominance of neutral carbohydrates other than uronic acids (91.1–94.0 mol % vs. 5.9–8.8 mol %). The galactose content was maximal in *F. camschatica* (48.8 mol %) and minimal in *F. ulmaria* (40.5 mol %). Also, high amounts of glucose were observed in the range of 23.4–36.4 mol %. Arabinose and mannose were important components of the WSP fraction with contents of 4.2–10.5 mol % and 6.0–7.0 mol %, respectively. The minor monosaccharides were fucose (0.2–0.9 mol %), xylose (0.4–2.7 mol %) and rhamnose (0.8–3.1 mol %).

**Figure 2:**
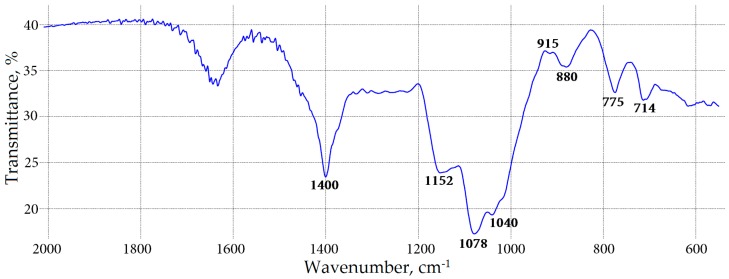
FT-IR spectra of water-soluble polysaccharide from *F. denudata* flowers.

The FT-IR spectra of the WSP fractions of four meadowsweet floral teas were very close to each other and similar to those for (arabino)galactan polysaccharides [30]. The FT-IR spectrum of WSP of *F. denudata* is shown in Figure 2. It contains strong bands assigned to stretching vibrations of pyranose and furanose rings (1152, 1078, 1040 cm^−1^), bands of C_1_-H_β_ deformation vibrations in the “anomeric region” (915, 880, 775, 714 cm^−1^) and less intensive bands of carboxylate groups (1400, 1650 cm^−1^).

The data allowed us to conclude that the WSP of *Filipendula* flowers are generally galactans and/or arabinogalactan with an admixture of glucans of the starch type and galacturonans as minor components. The known data on the biological activity of galactan-type polymers of plant origin suggest their effectiveness as immune modulators due to their influence on the complement system and phagocytosis [31]. For further structural investigations, *Filipendula* flower WSP should be subjected to bioactivity assessments such as immune modulatory testing to determine their role in the therapeutic effectiveness of meadowsweet teas.

### 2.6. Biological Activity of Meadowsweet Floral Teas Decoctions

#### 2.6.1. Inhibitory Effect on Amylase, α-Glucosidase and AGEs Formation

The investigation of the influence of meadowsweet teas on amylase demonstrated their ability to inhibit enzymatic activity with the highest IC_50_ value for *F. denudata* tea (IC_50_ 74.80 μg/mL) and the lowest value for *F. camschatica* (IC_50_ > 100 μg/mL) (Table 6). 

A similar trend was seen in the α-glucosidase inhibition experiments. *F. stepposa* was the most active meadowsweet tea (IC_50_ 71.35 μg/mL) and *F. camschatica* displayed no inhibition of α-glucosidase activity up to a concentration >100 μg/mL.

Separate compounds used as references in both experiments were either inactive, such as spiraeoside or methyl salicylate (IC_50_ > 100 μg/mL), or strongly active, such as a rugosin D. The latter compound was more active than acarbose, a known amylase and α-glucosidase inhibitor, used us as the positive control. The results regarding the inhibitory activity of these teas against AGE production displayed potent activity of *F. denudata*, *F. stepposa* and *F. ulmaria* teas with 52.11%, 61.18% and 32.90% percentage inhibition for solutions with a concentration of 1 mg/mL. The most active compound was rugosin D with an inhibition value of 69.72% at 0.5 mg/mL in contrast to inactive spiraeoside and methyl salicylate. Experiments with WSP of *Filipendula* flowers showed no inhibitory effects on the activity of enzymes and AGE formation.

Summarizing the results of the inhibitory potentials of the meadowsweet teas on the activity of amylase, α-glucosidase and AGE formation, we can confirm the potential of *Filipendula* plants as antidiabetic agents. The use of phenolic compounds as inhibitors of amylase and α-glucosidase in the treatment of diabetic disorders is related to their ability to reduce the postprandial blood glucose level [32]. A high level of the blood glucose leads to the accumulation of AGE, leading to the development of pathological conditions related to diabetes (nephropathy and neuropathy, cardiovascular disease and atherosclerosis) [33] Thus, the search for compounds that provide a combined positive effect on these negative processes is important.

Rosaceous plants are a known source of extracts with amylase inhibiting activity. Some species inhibit amylase, like *Rosa gallica*, *R. suavissimus*, *Rubus idaeus* and *Sorbus aucuparia* with IC_50_ values of 20–140 μg/mL [34]. The main chemical factors providing this activity are ellagitannins, which have demonstrated strong inhibition of amylase [35]. The potential of flavonoids as amylase inhibitors has been discussed previously [36], with a wide range of activity from inactive (or weakly active) to moderate. Close relationships have been observed between the ellagitannin content and the ability of crude plant extracts to inhibit α-glucosidase activity [37]. Despite this, only a few reports have discussed the inhibitory activity of rosaceous plants and ellagitannins on AGE formation in vitro [38]; the tannins of two *Chamaerhodos* species have been found to prevent AGE formation with IC_50_ values of 91–250 μM [39]. The anti-diabetic potential of *Filipendula* plants is discussed in this work and the results indicate no contradictions with known information about the bioactivity of Rosaceous plants and ellagitannins as a whole. 

#### 2.6.2. Antioxidant Activity

**Figure 3:**
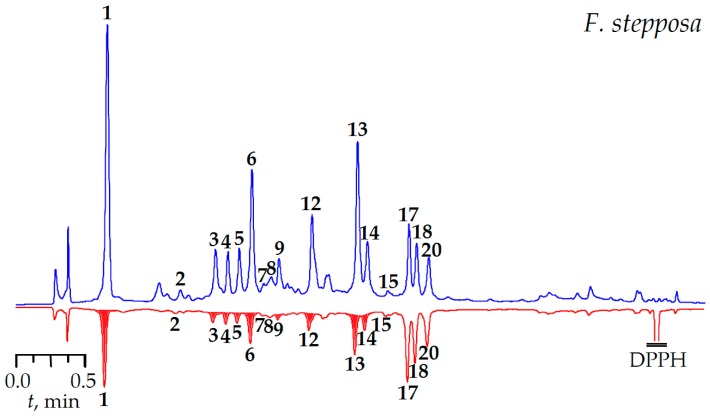
DPPH-MC-RP-HPLC-UV chromatograms of *F. stepposa* floral tea at 270 nm before (blue) and after (red) reaction with DPPH^•^-radicals. Compounds: **1**—gallic acid; **2**—protocatechuic acid; **3**—tellimagrandin I_1_; **4**—rugosin B_1_; **5**—tellimagrandin I_2_; **6**—rugosin B_2_; **7**—caffeic acid; **8**—1,3-di-*O*-caffeoylquinic acid; **9**—rugosin E_1_; **10**—rugosin E_2_; **11**—tellimagrandin II; **12**—rugosin D; **13**—ellagic acid; **14**—quercetin-3-*O*-β-d-glucoside (isoquercitrin); **15**—quercetin-3-*O*-α-l-arabinoside (avicularin); **16**—quercetin-3-*O*-β-d-glucuronide (miquelianin); **17**—quercetin-3-*O*-α-l-rhamnoside (quercitin); **18**—quercetin-4′-*O*-β-d-glucoside (spiraeoside); **19**—kaempferol-3-*O*-α-l-rhamnoside (afzelin); **20**—kaempferol-4′-*O*-β-d-glucoside

A preliminary characterization of antioxidant potential was carried out using the DPPH-MC-RP-HPLC-UV procedure, involving HPLC separation of samples pre-treated with an excess of DPPH^•^, followed by comparison with the HPLC chromatogram of an untreated sample. The reaction between an antioxidant and radical results in the oxidation of the antioxidant, which leads to a decrease in the corresponding peak areas in the chromatograms. The comparison of the HPLC chromatograms of untreated and radical-treated samples allows for the determination of the most active compounds. The chromatograms of the *F. stepposa* tea decoction spiked with DPPH^•^ radicals are shown in Figure 3, and present obviously reduced peak areas for some compounds in comparison with untreated samples.

Numerous compounds demonstrated a visible reduction of the peak area after spiking with DPPH^•^ radicals (gallic acid, peak 1; tellimagrandin I_1_, peak 3; rugosin B_1_, peak 4; tellimagrandin I_2_, peak 5; rugosin B_2_, peak 6; rugosin E_1_, peak 9; rugosin D, peak 12; ellagic acid, peak 13; isoquercitrin, peak 14). The majority of ellagitannins possess antioxidant activity and they may be considered as the major active compounds, while only one flavonoid, isoquercetin, revealed a high antioxidant potential. The results show the leading role of ellagitannins in free radical scavenging of meadowsweet teas.

The antioxidant properties of four *Filipendula* decoctions were evaluated using various tests: the 2,2-diphenyl-1-picrylhydrazyl radical (DPPH^•^) scavenging assay, the 2,2′-azino-bis(3-ethyl-benzthiazoline-6-sulphonic acid) radical (ABTS^•+^) scavenging assay, the Br^•^-radical scavenging activity and the carotene bleaching assay (CBA). All experiments included the determination and comparative estimation of the same antioxidant factors for spiraeoside, rugosin D and methyl salicylate, the representatives of the main classes of compounds dominating in the investigated tea samples; Trolox was used as a reference compound. As can be observed in Table 7, *F.* *stepposa* tea was the most active antioxidant in all assays, followed by *F. denudata*, *F. ulmaria* and *F. camschatica* teas. 

Early information about the quantitative content of the individual compounds in the meadowsweet teas allowed us to associate the significant antioxidant potential of *F*. *denudata* decoction with the highest ellagitannin content.

Decoctions of *F. stepposa* and *F. denudata* exhibited a similar ability to inactivate DPPH^•^ and ABTS^•+^ free radicals and demonstrated the most pronounced scavenging activity of these radicals. Less pronounced activity was observed for *F. ulmaria* and *F. camschatica* teas in both tests. The scavenging value of the *F. stepposa* decoction against bromine radicals was the highest (259.64 mg/g), while the lowest value was observed for *F. camschatica* tea (111.89 mg/g). The examination of the influence of meadowsweet teas on the oxidative destruction of β-carotene in the oleic acid-DMSO-H_2_O_2_ system demonstrated a high degree of antioxidant activity for *F. stepposa*, *F. denudata* and *F. ulmaria* tea samples with IC_50_ values of 3.53, 4.11 and 4.55 μg/mL.

The efficiency of *F. camschatica* in this assay was the lowest (IC_50_ 21.16 μg/mL). The investigation into the antioxidant activity of individual reference compounds demonstrated a wide range of activity. Rugosin D was the most active compound in all assays, while the least active compound was methyl salicylate.

Previously, the antioxidant properties of several *Filipendula* extractions were analyzed. The data on the antioxidant properties of the aqueous fraction from the aerial parts of *F. ulmaria* from Lithuania revealed lower efficiency in the scavenging of DPPH^•^ and ABTS^•+^ free radicals (IC_50_ 410 and 730 μg/mL, respectively) [40]. A possible reason for this phenomenon is the high contents of leaves and stems vs. flowers in a sample of the aerial parts. Also, a reduced content of ellagitannins in leaves reflects their lower effectiveness in radical scavenging [21]. The methanolic extract of *F. ulmaria* aerial parts collected in Serbia revealed effective scavenging of DPPH^•^ and ABTS^•+^ radicals, i.e., 16.41 μg/mL and 36.75 μg/mL, respectively [41]. The methanolic extract from flowers of *F. vulgaris* (*F. hexapetala*; Serbian origin) has been shown to be effective in the DPPH^•^ assay with an IC_50_ of 8.25 μg/mL [42]. In the ABTS^•+^ assay, the methanolic extract of the aerial parts of the same plant species demonstrated high efficiency in the scavenging of free radicals with an IC_50_ value of 34.52 μg/mL, whereas the scavenging ability of the aerial parts against DPPH^•^ radicals was lower than in flowers, i.e., 13.47 μg/mL [43]. The scavenging value of the aqueous fraction from the leaves and stems of *F. vulgaris* against DPPH^•^ radicals was less pronounced and amounted to an IC_50_ of 650 μg/mL [44]. 

The results obtained in the present study indicated a greater antioxidant effect of *F. stepposa*, *F. denudata* and *F. ulmaria* tea decoctions, indicating the effectiveness of meadowsweet tea for the regulation of antioxidant status in humans.

#### 2.6.3. Anti-Complement Activity of *Filipendula* Flower Water-Soluble Polysaccharides

**Figure 4:**
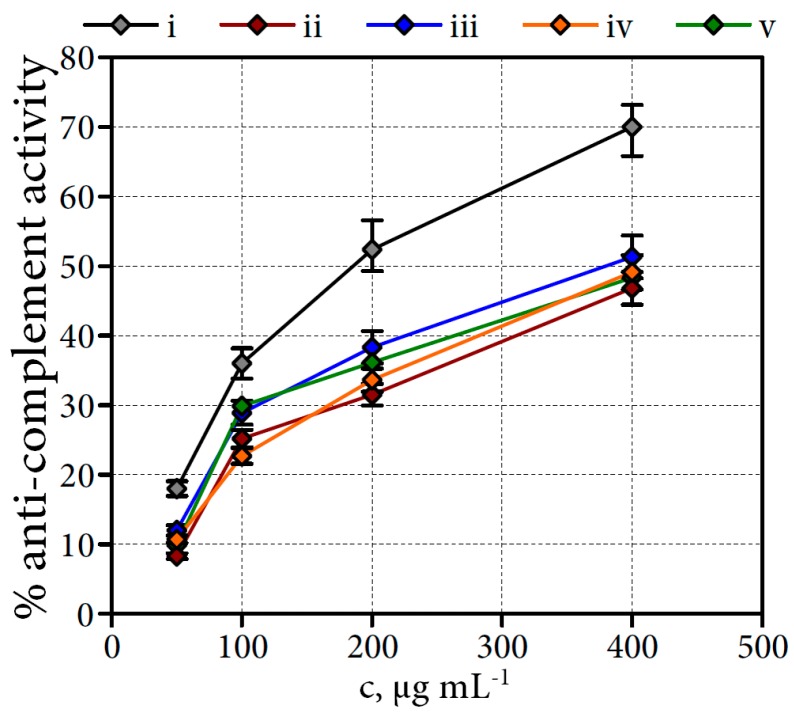
Anti-complement activity of crude water-soluble polysaccharide fractions (WSPS) from four meadowsweet floral teas. Samples: i—polysaccharide MPP′-2 from *Mentha piperita* (positive control); ii—WSPS from *F. camschatica* flowers; iii—WSPS from *F. denudata* flowers; iv—WSPS from *F. stepposa* flowers; v—WSPS from *F. ulmaria* flowers.

The test used for the biological activity investigation of WSP was the anti-complement activity test, which is an in vitro test to assess the ability of polysaccharides to interact with the complement cascade reaction [45]. Four crude WSP fractions from *Filipendula* flowers were tested for their anti-complement activity. As illustrated in Figure 4, all WSP fractions were less effective than the bioactive polysaccharide MPP′-2 from *Mentha piperita* used as a positive control. WSP from *F. denudata* flowers were found to have highest activity, i.e., 51.3% at a concentration 400 μg·mL^−1^ (vs. 70% for the polysaccharide MPP′-2). 

Polysaccharides from *F. camschatica*, *F. stepposa* and *F. ulmaria* flowers activated the complement system, to a similar extent as the WSP from *F. denudata* flowers, and are therefore considered to be biologically active in the same manner. The activity modes for all polysaccharide samples were dose-dependent. 

Several anti-complement galactans have been isolated from plants. Examples of bioactive polysaccharides are (arabino)galactans from *Angelica acutiloba* [46], *Plantago major* [47] and *Calendula officinalis* [48]. This is the first report of the anti-complement activity of WSP from meadowsweet plants.

The complement system is a part of the immune system consisting of different serum proteins activated by means of a cascade mechanism. Activation of the complement system is important in initiating inflammatory processes and in inducing leukocyte activation and the degranulation of basophils and mast cells [45]. The present results demonstrate the potent anti-complement activity of the crude WSP fractions of *Filipendula* flowers, and indicate their potent anti-inflammatory and immune-modulating ability.

## 3. Materials and Methods

### 3.1. Plant Materials and Chemicals

The samples of meadowsweet flowers were collected in the appropriate flowering period: *F. camschatica* (Pall.) Maxim. (*syn*. *F. camtschatica* (Pall.) Maxim., *F. kamtschatica* auct.)—Zaozernyi (Kamchatka Krai; 14.VII.2012, 53°01′55″ N, 158°78′01″ E, voucher specimen No. FRo/ae-31/15-08/0712); *F. denudata* (J.Presl & C.Presl) Fritsch (*syn. F. ulmaria* subsp. *denudata* (J.Presl & C.Presl) Hayek)—Kremeno (Gatchina District, Leningrad Oblast; 11.VII.2012, 59°35′21′′ N, 30°29′21′′ E, voucher specimen No. FRo/ae-24/16-08/0712); *F. stepposa* Juz. (*syn*. *F. ulmaria* subsp. *picbaueri* (Podp.) Smejkal)*—*Suzun (Suzunsky District, Novosibirsk Oblast; 5.VII.2012, 53°77′08′′ N, 82°28′07′′ E, voucher specimen No. FRo/ae-22/14-08/0712); *F. ulmaria—*Yakutsk (Yakutskii region, Sakha (Yakutia) Republic; 14.VI.2015, 62°9′57″ N, 129°36′42″ E, voucher specimen No. FRo/ae-03/18-41/0615). The species was determined by Prof. T.A. Aseeva (IGEB SB RAS, Ulan-Ude). The flowers were dried in a convective drying oven UT-4610 (Ulab, Sankt-Petersburg, Russia) at 40 °C (20–24 h) up to the humidity level 9%–12%. The flowers were grounded in an analytical mill A11 basic (IKA^®^-WerkeGmbH&Co.KG, Staufen, Germany) and then sieved using sieving machine ERL-M1 (Zernotekhnika, Moscow, Russia) up to an average particle diameter of 0.5 mm. The chemicals were purchased in Biosupplies Australia Ply Ltd. (Victoria, Australia)—Yariv reagent kit; Extrasynthese (Lyon, France)—quercetin-3-*O*-β-d-glucoside (isoquercitrin), quercetin-3-*O*-β-d-glucuronide (miquelianin), quercetin-3-*O*-α-l-rhamnoside (quercitin), quercetin-4′-*O*-β-d-glucoside (spiraeoside); Sigma-Aldrich (St. Louis, MO, USA)—acarbose, α-amylase from *Aspergillus oyzae* (30 U/mg), 4-aminoantipyrin, anthrone, arabinose, 2,2′-azino-bis(3-ethylbenzothiazoline-6-sulfonic acid) diammonium salt (ABTS), bovine serum albumin (BSA), Bradford reagent, caffeic acid, β-carotene, citric acid, 1,3-di-*O*-caffeoylquinic acid, 3,5-dimethylphenol, 2,2-diphenyl-1-picrylhydrazyl radical (DPPH^•^), dimethylsulphoxide (DMSO), ellagic acid, fructose, galactose, galacturonic acid, gallic acid, α-glucosidase from *Saccharomyces cerevisiae* (type I, 10 U/mg), glucose, glucose oxidase from *Aspergillus niger* (type II, 15,000 U/g), glucuronic acid, hydrogen peroxide (*ca.* 30%), kaempferol-3-*O*-α-l-rhamnoside (afzelin), lithium perchlorate, malic acid, mannose, methyl salicylate, *p*-nitrophenyl α-d-glucopyranoside, oleic acid, oxalic acid, perchloric acid (≥70%, 99.999% trace metals basis), peroxidase from horseradish (150 U/mg), protocatechuic acid, starch soluble, quercetin, quercetin-3-*O*-α-l-arabinoside (avicularin), quinic acid, resorcinol, rhamnose, sodium persulphate, succinic acid, sucrose, tartaric acid, trichloroacetic acid (TCA), Tween^®^ 80, xylose. Kaempferol-4′-*O*-β-d-glucoside, tellimagrandins I and II, rugosins B_1,_ B_2_, E_1_, E_2_ and D were isolated previously from *F. ulmaria* [21]. Equipment used for UV-Vis spectrophotometry was SF-2000 UV-Vis-spectrophotometer (OKB Specter; St. Peterburg, Russia); analyt. MC-HPLC—microcolumn chromatograph MiLiChrom A-02 (Econova; Novosibirsk, Russia); mineral analysis—ELAN DRC II mass-spectrometer (PerkinElmer, Inc.; Shelton; CT; USA); GC/MS—6890 N gas chromatograph coupled to a 5973 N mass selective/quadrupole detector (Agilent Technologies; Santa Clara, CA, USA); fluorimetry—Fluorat 02-5M fluorimeter (Lumex; Saint Petersburg, Russia); coulometry—Expert-006 potentiostat (Econix-Expert; Moscow, Russia).

### 3.2. Organoleptic and Nutritional Analysis

#### 3.2.1. Decoction Preparation

The sample of dried milled herb (1 g) in thermostable flat-bottomed flask (250 mL) was mixed with distilled water (100 mL), reflux condenser was attached with flask and then boiled on heater plate at 10 min. The mixture was left to stand at room temperature for 15 min, and then filtered throw the 0.45 μm PTFE filter under reduced pressure into the volumetric flask (100 mL) and volume was filled with distilled water up to 100 mL.

#### 3.2.2. Crude Composition

Organoleptic parameters (color, odor, taste) of meadowsweet teas were determined accordingly AHPA guidance on Organoleptic Analysis [49]. Extractives and ash were determined accordingly WHO recommendations [50]. The protein content was estimated by Bradford method using BSA as a reference substance [51]. The lipid content was determined by extracting a known weight of dried meadowsweet tea with chloroform–methanol mixture (4:1) using Soxhlet apparatus. Carbohydrate content was determined with spectrophotometric phenol–sulphuric acid method [52]. Energy was calculated according to the following equation: Energy (kcal) = 4 × (g protein + g carbohydrate) + 9 × (g lipids). Free amino acids content was determined with ninhydrin method [50].

#### 3.2.3. Free Sugars Composition

Free sugars were determined by a Milichrom A-02 microcolumn HPLC system, using Separon 5-NH_2_ column (1 mm × 60 mm, Ø 1 μm; Tessek Ltd.; Prague, Czech Republic), column temperature was 20 °C. Mobile phase was acetonitrile–water 75:25. The injection volume was 1 μL, and elution was at 100 μL/min. Detector wavelength was 190 nm. Decoctions of meadowsweet floral herb teas prepared accordingly a protocol described in Section 3.2.1 were filtered through a 0.22 μm PTFE syringe filter before injection into the HPLC system for analysis. Stock solutions of standards were made by accurately weighing 10 mg of fructose, galactose, glucose and saccharose and dissolving it in 10 mL of water in a volumetric flask. The appropriate amounts of stock solutions were diluted with water in order to obtain standard solutions containing 0.25–1.00 mg/mL. As all the compounds used for quantification were well-separated in experiment conditions mixtures of standards were analyzed. Prepared solutions were stored at 4 °C for no more than 10 h. The results are presented as mean values ± SD (standard deviations) of the three replicates.

#### 3.2.4. Organic Acids Composition

Organic acids were determined by a Milichrom A-02 microcolumn HPLC system, using a ProntoSIL-120-5-C18 AQ column (1 mm × 70 mm, Ø 5 μm; Metrohm AG; Herisau, Switzerland), column temperature was 35 °C. Eluent A was 0.2 M LiClO_4_ in 0.01 M HClO_4_ and eluent B was acetonitrile. The injection volume was 1 μL, and elution was at 50 μL/min. Gradient programme: 0–20 min, 1% B; 20–25 min, 1%–10% B. Detector wavelength was 210 nm. Decoctions of meadowsweet floral herb teas prepared accordingly a protocol described in Section 3.2.1 were filtered through a 0.22 μm PTFE syringe filter before injection into the HPLC system for analysis. Stock solutions of standards were made by accurately weighing 2 mg of citric, malic, oxalic, quinic, succinic and tartaric acids and dissolving it in 10 mL of water in a volumetric flask. The appropriate amounts of stock solutions were diluted with water in order to obtain standard solutions containing 0.10–0.50 mg/mL. As all the compounds used for quantification were well-separated in experiment conditions mixtures of standards were analyzed. Prepared solutions were stored at 4 °C for no more than 10 h. The results are presented as mean values ± SD (standard deviations) of the three replicates.

#### 3.2.5. Amino Acids Composition

Amino acids were determined as phenylthiocarbamyl derivatives using a Milichrom A-02 microcolumn HPLC system with a Nucleosil-100-C18 column (2 mm × 75 mm, Ø 5 μm; Macherey-Nagel GmbH & Co. KG; Düren, Germany), column temperature was 60 °C. Eluent A was mixture of 1 M CH_3_COONH_4_/H_3_PO_4_ (pH 5.25) and water (5:95) and eluent B was mixture of 1 M CH_3_COONH_4_/H_3_PO_4_ (pH 6.50), acetonitrile and water (5:35:60). The injection volume was 2 μL, and elution was at 150 μL/min. Gradient programme: 0–6.6 min, 0%–12% B; 6.6–21.3 min, 12%–65% B; 21.3–30 min, 100% B. Detector wavelength was 246 nm. Decoctions of meadowsweet floral teas prepared accordingly a protocol described in Section 3.2.1 were filtered through a 0.22 μm PTFE syringe filter before derivatization procedure using Scholtze method [53]. Sigma standard mixture of 22 amino acids (Cat. No. 094165, ≥99%) was used as a reference mixture. As all the compounds used for quantification were well-separated in experiment conditions mixtures of standards were analyzed. Prepared solutions were stored at 4 °C for no more than 72 h. The results are presented as mean values ± SD (standard deviations) of the three replicates.

#### 3.2.6. Minerals Composition

The content of Ca, Cr, Co, Cu, Fe, Mg, Mn, Mo, Ni, Se, Zn were determined in meadowsweet floral tea decoctions by inductively coupled plasma mass spectrometry (ICP-MS) using ELAN DRC II mass-spectrometer (PerkinElmer, Inc., Shelton, CT, USA). The protocol used has been described elsewhere [54]. Decoctions of meadowsweet floral herb teas prepared accordingly a protocol described in Section 3.2.1 were filtered through a 0.22 μm PTFE syringe filter before analysis. The results are presented as mean values ± SD (standard deviations) of the three replicates.

### 3.3. MC-RP-HPLC Quantification of Phytochemicals in Meadowsweet Teas

MC-RP-HPLC experiments were performed on an MiLiChrom A-02 microcolumn chromatograph coupled with a UV-detector, using a ProntoSIL-120-5-C18 AQ column (1 × 50 mm, Ø 1 μm; Metrohm AG; Herisau, Switzerland); the column temperature was 35 °C. Eluent A was 0.2 M LiClO_4_ in 0.01 M HClO_4_ and eluent B was acetonitrile. The injection volume was 1 μL, and elution was at 600 μL/min. Gradient program: 0–1.9 min, 7%–22% B; 1.9–2.2 min, 22%–25% B; 2.2–3.0 min, 25%–27% B; 3.0–4.3 min, 27%–100% B; 4.3–5.0 min, 100% B. UV-detector wavelengths were 270 nm. Decoctions of meadowsweet floral herb teas prepared accordingly a protocol described in Section 3.2.1 were filtered through a 0.22 μm PTFE syringe filter before injection into the HPLC system for analysis. Stock solutions of standards were made by accurately weighing 1 mg of gallic acid, protocatechuic acid, caffeic acid, 1,3-di-*O*-caffeoylquinic acid, ellagic acid, methyl salicylate, tellimagrandins I and II, rugosins B_1,_ B_2_, E_1_, E_2_, D, quercetin-3-*O*-β-d-glucoside (isoquercitrin), quercetin-3-*O*-α-l-arabinoside (avicularin), quercetin-3-*O*-β-d-glucuronide (miquelianin), quercetin-3-*O*-α-l-rhamnoside (quercitin), quercetin-4′-*O*-β-d-glucoside (spiraeoside), kaempferol-3-*O*-α-l-rhamnoside (afzelin), kaempferol-4′-*O*-β-d-glucoside and quercetin and dissolving it in 20 mL of methanol/DMSO in a volumetric flask. The appropriate amounts of stock solutions were diluted with methanol in order to obtain standard solutions containing 0.25–1.00 mg/mL. As all the compounds used for quantification were well-separated in experiment conditions mixtures of standards were analyzed. Prepared solutions were stored at 4 °C for no more than 72 h. The results are presented as mean values ± SD (standard deviations) of the three replicates.

### 3.4. Essential Oil Analysis

Dry flowers of *Filipendula* species (300 g) were subjected to hydrodistillation in Clevenger-type apparatus for 150 min, which gave the essential oil. GC/MS analysis was performed on an Agilent 6890 N gas chromatograph coupled to a Agilent Technologies 5973 N mass selective/quadrupole detector using a fused capillary column HP-5MS (30 m × 0.25 mm, film thickness 0.50 μm, 5% diphenyl- and 95% dimethylpolysiloxane stationary phase). Splitless injection of 0.2 μl sample solution in hexane (~1%) was performed using an injection port temperature of 250 °C. The carrier gas was helium at a flow of 1.0 mL/min. The column temperature was programmed from 150 to 250 °C at 2.0 °C/min. The ion source temperature was 230 °C. The EIMS spectra (70 eV) were obtained in the scan mode in *m*/*z* range 41–450 a.u.m. Identification of compounds was made by comparison of their retention times with those of analytical standards of available terpenoids, on comparison of mass spectra with those found in the literature, and the mass spectrometry data bank (NIST 05) and computer search of the Wiley library. For quantification purposes, relative area percentages by FID were used.

### 3.5. Polysaccharide Analysis

The sample of dried milled herb (100 g) was added to distilled water (10 L), heated on a boiled water bath (1 h) and af ter cooling to room temperature water extract was filtered under reduced pressure and concentrated down *in vacuo* to 200 mL. The concentrated residue was mixed with 95% ethanol (1:5) and after 2 h the precipitate was centrifuged at 3000 *g*. The crude polysaccharide fraction was redissolved in 200 mL of water. The Sevag method [55] was used for deproteinisation, and was followed by dialysis for 48 h against distilled water using dialysis tubes with an MW-cut off of 2 kDa (Sigma-Aldrich, St. Louis, MO, USA). The non-dialysed part was loaded on to a KU-2-8 cation-exchange resin column (H^+^-form, 200 g; Closed Joint-Stock Company Tokem, Kemerovo, Russia) which was eluted with 2 L of distilled water. Eluate was concentrated *in vacuo* up to 200 mL and then liophylized. WSPF obtained were off-white powders. Total carbohydrate content was determined with the spectrophotometric anthrone-sulphuric acid method [56]; content of uronic acids was estimated by the 3,5-dimethylphenol method calculated as galacturonic acid [57]; and the proteins were determined by the Bradford method using Coomassie G250 [51]. Reactions of WSPF solutions with iodine, resorcinol and Yariv reagent were performed accordingly [58,59,60]. IR spectra were registered in a spectral range of 4000–600 cm^−1^ using a FT-801 Fourier-transform infrared spectrometer (Simex, Novosibirsk, Russia) coupled with a single reflection ATR device. The hydrolysis procedure and HPLC analysis conditions of the released products were as described by us previously [61].

### 3.6. Biological Activity

#### 3.6.1. Amylase Inhibitory Activity

Amylase inhibitory activity was assayed according to a previously published spectrophotometric protocol [62]. Sample solution in DMSO (10 μL), 30 μL of phosphate buffer (pH 5.0) and 10 μL of amylase from *Aspergillus niger* (3 U/mL) were incubated for 20 min at 45 °C. Then 10 μL of 2% starch solution, 40 μL of phosphate buffer (pH 5.0) and 100 μL of the reagent were added and incubated for 30 min at 50 °C. Absorbance was measured at 510 nm. The reagent was a solution of K_2_HPO_4_ (0.8 mM), KH_2_PO_4_ (0.4 mM), phenol (220 mM), 4-aminoantipyrine (1.5 μM), glucose oxidase from *Aspergillus niger* (3 U/mL), and peroxidase from horseradish (0.3 U/mL) in deionized water. A 2% solution of acarbose was used as a positive control (PC), and water was used as a negative control (NC). The ability to inhibit amylase was calculated using the following equation: Inhibitory ability (%) = [(A_510_^NC^ − A_510_^PC^) – (A_510_^Sample^ − A_510_^PC^) / (A_510_^NC^ − A_510_^PC^)] × 100, where A_510_^NC^ is the absorbance of the negative control, A_510_^PC^ is the absorbance of the positive control and A_510_^Sample^ is the absorbance of the sample solution. The IC_50_ value is the effective concentration at which amylase activity was inhibited by 50%. Values are expressed as mean obtained from five independent experiments.

#### 3.6.2. α-Glucosidase Inhibitory Activity

The α-glucosidase inhibition assay was performed using spectrophotometric method [63]. α-Glucosidase from *Saccharomyces cerevisiae* was dissolved in phosphate buffer (pH 6.8) containing BSA (0.2%) up to 0.5 U/mL concentration. Solution (10 μL) of sample in phosphate buffer (pH 6.8) at varying concentrations (10–1000 μg/mL) was premixed with 490 μL of phosphate buffer (pH 6.8) and 250 μL 5 mM *p*-nitrophenyl α-d-glucopyranoside. After preincubating at 37 °C for 5 min, 250 μL α-glucosidase (0.4 U/mL) was added and incubated at 37 °C for 15 min. The reaction was terminated by the addition of 2000 μL Na_2_CO_3_ (200 mM). Absorbance was measured at 400 nm. A 2% solution of acarbose was used as a positive control (PC), and water was used as a negative control (NC). The ability to inhibit amylase was calculated using the following equation: Inhibitory ability (%) = [(A_400_^NC^ – A_400_^PC^) – (A_400_^Sample^ – A_400_^PC^)/(A_400_^NC^ – A_400_^PC^)] × 100, where A_400_^NC^ is the absorbance of the negative control, A_400_^PC^ is the absorbance of the positive control and A_400_^Sample^ is the absorbance of the sample solution. The IC_50_ value is the effective concentration at which α-glucosidase activity was inhibited by 50%. Values are expressed as mean obtained from five independent experiments.

#### 3.6.3. AGEs Formation Inhibitory Activity

The inhibitory effect on AGEs formation reaction was carried out by fluorimetric method of Matsuura et al. [64]. The reaction mixture consisted of 4 mg BSA in 400 μL 50 mM sodium phosphate buffer (pH 7.4), 80 μL 1 M glucose, and 20 μL of sample (meadowsweet tea decoction or pure compound in water or DMSO). The reaction solution was incubated at 60 °C for 48 h. The blank sample which contained no meadowsweet tea decoction or pure compound was kept at 4 °C until measurement. After cooling aliquots of 250 μL were transferred to 1.5 mL plastic tubes, then, 25 μL TCA was added to each tube and stirred. The supernatant was removed after centrifugation (15,000 rpm) at 4 °C for 4 min and AGEs-BSA precipitate was dissolved with 1 mL of sodium phosphate buffer (pH 7.4). This solution was monitored fluorimetrically in fluorescence intensity (ex. 360 nm, em. 460 nm). The ability to inhibit AGEs formation was calculated using the following equation: Inhibitory ability (%) = [1 − (F_S_/F_B_)] × 100, where F_S_ is the fluorescence of the sample, F_B_ is the fluorescence of the blank. Values are expressed as mean obtained from three independent experiments.

#### 3.6.4. DPPH-HPLC-UV Procedure

The DPPH-HPLC-UV procedure was realized as described previously [65]. Briefly, a sample of the meadowsweet tea decoction (100 μL) were added to DPPH^•^ radical solution in methanol (250 μL, 20 mg/mL). The mixture was shaken for few seconds and left to stand in the dark for 30 min at room temperature. Then the sample was filtered through a 0.22 μm membrane filter. The untreated sample was prepared by adding a sample of the meadowsweet tea decoction (100 μL) to methanol (250 μL). HPLC analysis was performed on as described in a Section 3.4.

#### 3.6.5. DPPH^•^ Radical Scavenging Activity

The DPPH^•^ radical scavenging activity (DPPH^•^) was assessed as described by Asker and Shawky [66]. 500 μL of a DPPH^•^ methanol solution (freshly prepared, 100 μg/mL) was added to 500 μL of sample solution (meadowsweet tea decoction or pure compound in methanol). After 15 min absorbance was measured at 520 nm. A 0.01% solution of gallic acid was used as a positive control (PC), and water was used as a negative control (NC). The ability to scavenge DPPH^•^ radicals was calculated using the following equation: Scavenging ability (%) = [(A_520_^NC^ − A_520_^PC^) – (A_520_^Sample^ − A_520_^PC^)/(A_520_^NC^ − A_520_^PC^)] × 100, where A_520_^NC^ is the absorbance of the negative control, A_520_^PC^ is the absorbance of the positive control and A_520_^Sample^ is the absorbance of the sample solution. The IC_50_ value is the effective concentration at which DPPH^•^ radicals were scavenged by 50%. Values are expressed as mean obtained from five independent experiments.

#### 3.6.6. ABTS^•+^ Radical Scavenging Activity

The ABTS^•+^ radical scavenging activity (ABTS^•+^) was measured using the method of Ding et al. [67]. ABTS was dissolved in water to a concentration of 7 mM. ABTS^•+^ radical cations were produced by reacting this ABTS^•+^ stock solution with 2.45 mM potassium persulphate (final concentration) and allowing the mixture to stand in the dark at room temperature for 12–16 h before use. The ABTS^•+^ radical cation solution was diluted with methanol to an absorbance of 0.70 at 734 nm and equilibrated at 30 °C. An aliquot of sample solution (500 μL) was mixed with 500 μL of diluted ABTS^•+^ radical cation solution. After reaction at 30 °C for 20 min, the absorbance was measured at 734 nm. A 0.01% solution of gallic acid was used as a positive control (PC), and water was used as a negative control (NC). The ability to scavenge the ABTS^•+^ radical cation was calculated using the following equation: Scavenging ability (%) = [(A_734_^NC^ – A_734_^PC^) – (A_734_^Sample^ – A_734_^PC^) / (A_734_^NC^ – A_734_^PC^)] × 100, where A_734_^NC^ is the absorbance of the negative control, A_734_^PC^ is the absorbance of the positive control and A_734_^Sample^ is the absorbance of the sample solution. The IC_50_ value is the effective concentration at which ABTS^•+^ radicals were scavenged by 50%. Values are expressed as mean obtained from five independent experiments.

#### 3.6.7. Br^•^ Radical Scavenging Activity

The bromine radical scavenging activity (Br^•^-SA) was determined using culometric method [68] with electrogenerated bromine radicals. Coulometric measurements were carried out using Expert-006 potentiostat with four-electrode two-compartment electrochemical cell. A bare platinum foil with 1 cm^2^ surface area was used as the working electrode, and a platinum wire separated from the anodic compartment with a semipermeable diaphragm—as the auxiliary electrode. A pair of polarized platinum electrodes was used for detection of the titration end-point (Δ*E* = 200 mV). Surface of platinum electrodes was cleaned by HNO_3_ and then rinsed thoroughly with double distilled water. Electrochemical generation of titrant was carried out from 0.2 M KBr in 0.1 M H_2_SO_4_ at a current density 5 mA∙cm^−2^ providing 100% current yield. Coulometric titration was carried out in a 50 mL cell containing 20.0 mL of supporting electrolyte. The generating circuit was switched on and a certain value of the indicator current was attained. Then an aliquot portion (50 μL) of meadowsweet tea decoction (or gallic acid, 1 mg/mL, as a reference compound) was added to the cell and timer was simultaneously started. The titration end-point was detected by the attainment of the initial value of the indicator current. The timer was stopped, and the generating circuit was turned off. The time of titration were used for the Br^•^-SA calculation that were expressed in units of quantity of electricity (Coulombs (C)) spent for titration on 100 mL of meadowsweet tea decoction. Finally the value of Br^•^-SA was calculated as a mg gallic acid equivalents per g extract. Values are expressed as mean obtained from five independent experiments.

#### 3.6.8. β-Carotene Bleaching Assay

β-Carotene bleaching assay (CBA) was performed in β-carotene–oleic acid–DMSO–H_2_O_2_ system [69]. Two milligrams of β-carotene, 16 mg of oleic acid, and 160 mg of Triton X-100 were dissolved in 50 mL of chloroform, and organic solvent was removed in a vacuum at 30 °С. The residue was solved in bidistilled water (50 mL); solution was removed to a volumetric flask (100 mL) and water was added until the total volume reached 100 mL. The reaction solutions was prepared in a tubes by mixing 50–250 μL of test solution, 500 μL DMSO, 500 μL β-carotene and 500 μL of 3% H_2_O_2_ solution. The total volume of reaction solution was reached by adding of bidistilled water up to 2 mL. The mixture was placed in a thermostat at 50 °С for 120 min. Absorbance was measured at 460 nm at 0 min and 120 min after incubation. The ability to protect β-carotene was calculated using the following equation: Inhibitory ability (%) = 100 − {[(A_0_ – A_120_)/A_0_] × 100}, where A_0_ is the absorbance after 0 min of incubation, A_120_ is the absorbance after 120 min of incubation. The IC_50_ value is the effective concentration allowed to protect 50% of β-carotene. Values are expressed as mean obtained from three independent experiments.

#### 3.6.9. Complement Fixation Test

The complement fixation test based on inhibition of hemolysis of antibody sensitized sheep red blood cells by the complement from human sera was used [70]. Veronal buffer/bovine serum albumin, serum, and sensitized sheep erythrocytes were the control of the medium, and the rhamnogalacturonan MPP′-2 from *Mentha piperita* was used as positive control [71]. The indicator system in the assay is inhibition of haemolysis induced by human complement. Samples showing inhibition in the assay is thus having a direct effect on the human immune system. Inhibition of lysis induced by the test samples was calculated by the formula: Inhibition (%) = [(A_C_ – A_S_)/A_C_] × 100, where A_C_ is the absorbance the control, A_S_ is the absorbance of the sample. Values are expressed as mean obtained from three independent experiments.

### 3.7. Statistical Analysis

Statistical analyses were performed using a one-way analysis of variance (ANOVA), and the significance of the mean difference was determined by Duncan’s multiple range test. Differences at *p* < 0.05 were considered statistically significant. The results are presented as mean values ± SD (standard deviations) of the three replicates.

## 4. Conclusions

The present study provides detailed data on the nutritional profile, phenolic, essential oil, water-soluble polysaccharide composition and bioactivity of four herbal products used in Siberia, namely *Filipendula camschatica*, *F. denudata*, *F. stepposa* and *F. ulmaria* floral teas, or meadowsweet teas in general. As far as we know, up to now, this is the first study to report data on these parameters for the studied products. Macronutrients were found in appropriate amounts in meadowsweet teas, with carbohydrates being the predominant component. Phenolic profiling of the meadowsweet teas revealed high contents in flavonols and ellagitannins. Interestingly, the meadowsweet teas were also found to be a source of bioactive volatiles such as salicyaldehyde and methyl salicylate, which are the components of the essential oils of *Filipendula* flowers. It should be noted that the water-soluble components were characterized by the presence of polymeric carbohydrates with a high content of galactose. The bioactivity data demonstrated the good ability of meadowsweet teas to inhibit amylase, α-glucosidase and AGE formation, and also expressed antioxidant properties. The anti-complement activity of the water-soluble polysaccharide fraction indicated their possible immune-modulating properties. The results highlight that meadowsweet can be considered as a new natural source of functional beverages due to the high content of health-promoting compounds, including anti-diabetic and antioxidant phenolics and immune-active polysaccharides.

## Figures and Tables

**Table 1:** Organoleptic, macronutrients characteristics, free sugars, organic acids, amino acids and minerals composition of four meadowsweet teas (mean ± SD) ^a^.

Parameter	*F. camschatica*	*F. denudata*	*F. stepposa*	*F. ulmaria*
Extractives ^b^	310 ± 12	390 ± 15	394 ± 15	374 ± 14
Organoleptic characteristics				
Color	Yellow	Dark yellow	Yellow	Pale yellow
Odor	Specific, sharp, methyl salicylate like	Specific, sweet	Specific, sweet, honey like	Specific, sweet
Taste	Bitterish, astringent	Bitterish, astringent	Bitterish, astringent	Bitterish, astringent
Macronutrients				
Carbohydrates ^b^	173.81 ± 5.04	132.05 ± 3.82	163.25 ± 4.87	147.11 ± 3.97
Protein ^b^	10.30 ± 0.35	8.57 ± 0.31	12.49 ± 0.40	9.73 ± 0.32
Lipids ^b^	<1.00	<1.00	<1.00	<1.00
Ash ^b^	54.59 ± 1.91	40.98 ± 1.59	34.40 ± 1.16	45.63 ± 1.59
Energy ^c^	0.75	0.57	0.71	0.64
Free sugars				
Fructose ^b^	tr.	tr.	tr.	tr.
Galactose ^b^	11.22 ± 0.30	8.07 ± 0.21	9.02 ± 0.25	9.74 ± 0.30
Glucose ^b^	66.31 ± 1.85	54.39 ± 1.63	67.31 ± 2.01	65.27 ± 2.02
Sucrose ^b^	15.61 ± 0.43	11.86 ± 0.35	12.08 ± 0.34	10.18 ± 0.32
Total free sugars ^b^	93.14	74.32	88.41	85.19
Organic acids				
Citric acid ^b^	5.84 ± 0.15	10.84 ± 0.25	8.24 ± 0.19	7.11 ± 0.17
Malic acid ^b^	6.52 ± 0.15	11.67 ± 0.29	10.30 ± 0.26	9.58 ± 0.24
Oxalic acid ^b^	9.62 ± 0.24	6.04 ± 0.14	4.93 ± 0.12	5.74 ± 0.12
Quinic acid ^b^	0.11 ± 0.00	tr.	tr.	tr.
Succinic acid ^b^	tr.	0.10 ± 0.00	tr.	tr.
Tartaric acid ^b^	tr.	0.35 ± 0.01	0.19 ± 0.00	0.27 ± 0.00
Total organic acids ^b^	22.09	29.00	23.66	22.70
Amino acids				
Alanine ^b^	0.89 ± 0.02	0.26 ± 0.00	0.62 ± 0.02	0.75 ± 0.02
Arginine ^b^	1.28 ± 0.04	0.32 ± 0.01	0.98 ± 0.03	0.73 ± 0.02
Asparagine ^b^	0.56 ± 0.01	0.39 ± 0.01	0.58 ± 0.02	0.26 ± 0.00
Aspartic acid ^b^	3.35 ± 0.07	0.90 ± 0.02	3.71 ± 0.08	2.03 ± 0.05
Cisteine ^b^	0.05 ± 0.00	0.01 ± 0.00	0.03 ± 0.00	0.01 ± 0.00
Glutamine ^b^	0.02 ± 0.00	0.10 ± 0.00	0.14 ± 0.00	0.08 ± 0.00
Glutamic acid ^b^	3.65 ± 0.09	0.68 ± 0.02	2.59 ± 0.06	1.48 ± 0.03
Glycine ^b^	1.26 ± 0.04	0.29 ± 0.00	0.84 ± 0.02	0.67 ± 0.02
Histidine ^b^	0.81 ± 0.02	0.08 ± 0.00	0.37 ± 0.01	0.35 ± 0.00
Isoleicine ^b^	0.97 ± 0.03	0.17 ± 0.00	0.65 ± 0.01	0.59 ± 0.02
Leucine ^b^	1.55 ± 0.04	0.72 ± 0.02	1.45 ± 0.03	1.04 ± 0.03
Lysine ^b^	1.44 ± 0.04	0.28 ± 0.00	0.77 ± 0.02	0.86 ± 0.02
Methionine ^b^	1.01 ± 0.02	0.12 ± 0.00	0.40 ± 0.01	0.14 ± 0.00
Phenyalanine ^b^	0.89 ± 0.03	0.23 ± 0.00	0.57 ± 0.01	0.73 ± 0.01
Proline ^b^	0.77 ± 0.02	0.18 ± 0.00	0.85 ± 0.02	0.72 ± 0.01
Serine ^b^	1.43 ± 0.04	0.31 ± 0.01	0.78 ± 0.02	0.69 ± 0.01
Treonine ^b^	0.75 ± 0.01	0.14 ± 0.00	0.38 ± 0.01	0.58 ± 0.01
Tyrosine ^b^	0.65 ± 0.01	0.11 ± 0.00	0.02 ± 0.00	0.42 ± 0.01
Valine ^b^	1.25 ± 0.03	0.32 ± 0.01	0.71 ± 0.01	0.76 ± 0.02
Total amino acids ^b^	22.58	5.61	16.44	12.89
Minerals				
Calcium ^d^	396.29 ± 28.93	295.84 ± 23.08	239.42 ± 22.51	273.72 ± 24.09
Chromium ^d^	0.16 ± 0.01	0.23 ± 0.02	0.29 ± 0.02	0.20 ± 0.02
Cobalt ^d^	0.11 ± 0.01	0.05 ± 0.00	0.04 ± 0.00	0.05 ± 0.00
Copper ^d^	4.83 ± 0.44	5.31 ± 0.45	4.38 ± 0.40	4.74 ± 0.42
Iron ^d^	4.84 ± 0.42	4.70 ± 0.40	5.06 ± 0.41	11.12 ± 1.01
Magnesium ^d^	4923.48 ± 428.34	3266.55 ± 264.59	2726.8 ± 245.41	2730.73 ± 251.23
Manganese ^d^	2.78 ± 0.22	2.69 ± 0.22	1.21 ± 0.11	6.30 ± 0.60
Molybdenum ^d^	0.03 ± 0.00	0.08 ± 0.01	0.01 ± 0.00	0.05 ± 0.00
Nickel ^d^	0.33 ± 0.02	1.24 ± 0.10	1.43 ± 0.12	1.28 ± 0.11
Selenium ^d^	0.34 ± 0.03	0.01 ± 0.00	0.16 ± 0.01	0.26 ± 0.02
Zinc ^d^	14.51 ± 1.03	16.14 ± 1.48	11.88 ± 1.01	12.55 ± 0.99

^a^ standard brewing—1 g plant material/100 mL water; ^b^ mg/100 mL decoction; ^c^ kcal/100 mL decoction; tr.—traces amounts (<limit of quantification); ^d^ μg/100 mL decoction.

**Table 2:** General phytochemical characteristics of four meadowsweet teas, mg/100 mL decoction (±SD).

Parameter	*F. camschatica*	*F. denudata*	*F. stepposa*	*F. ulmaria*
Flavonoids	18.34 ± 0.42	50.92 ± 1.22	52.25 ± 1.35	55.31 ± 1.43
Tannins	51.83 ± 1.50	114.81 ± 3.55	120.18 ± 3.72	100.88 ± 3.21
Catechins	9.57 ± 0.39	1.27 ± 0.05	tr.	5.69 ± 0.24
Proanthocyanidins	4.64 ± 0.15	2.15 ± 0.06	5.14 ± 0.16	3.83 ± 0.12
WS-Polysaccharides	21.39 ± 0.56	24.51 ± 0.61	30.47 ± 0.94	23.81 ± 0.59

tr.—traces amounts (<limit of quantification).

**Table 3:** Content of the main phenolics in four meadowsweet tea decoctions, mg/100 mL decoction (±SD).

Compound	*F. camschatica*	*F. denudata*	*F. stepposa*	*F. ulmaria*
Flavonoids				
Kaempferol-3-*O*-α-l-rhamnoside	tr.	2.27 ± 0.07	2.78 ± 0.07	0.87 ± 0.02
Kaempferol-4′-*O*-β-d-glucoside	5.43 ± 0.14	4.70 ± 0.14	6.50 ± 0.19	5.71 ± 0.12
Quercetin	0.40 ± 0.01	1.71 ± 0.04	0.72 ± 0.02	1.20 ± 0.03
Quercetin-3-*O*-β-d-glucoside	0.74 ± 0.02	4.95 ± 0.14	10.18 ± 0.30	6.92 ± 0.16
Quercetin-3-*O*-α-l-arabinoside	0.76 ± 0.02	5.96 ± 0.17	4.21 ± 0.13	2.60 ± 0.05
Quercetin-3-*O*-α-l-rhamnoside	0.29 ± 0.01	1.18 ± 0.03	9.50 ± 0.27	3.92 ± 0.09
Quercetin-4′-*O*-β-d-glucoside	6.43 ± 0.17	28.50 ± 0.71	17.91 ± 0.45	17.53 ± 0.42
Quercetin-3-*O*-β-d-glucuronide	0.46 ± 0.01	tr.	tr.	tr.
Subtotal	14.51	49.27	51.80	38.75
Ellagitannins				
Tellimagrandin I_1_	1.32 ± 0.03	7.61 ± 0.19	9.30 ± 0.25	4.56 ± 0.10
Tellimagrandin I_2_	2.28 ± 0.06	6.58 ± 0.19	10.65 ± 0.29	6.71 ± 0.15
Tellimagrandin II	0.59 ± 0.02	2.27 ± 0.06	2.73 ± 0.07	2.02 ± 0.04
Rugosin B_1_	2.42 ± 0.07	8.24 ± 0.25	7.69 ± 0.19	5.35 ± 0.12
Rugosin B_2_	2.92 ± 0.09	12.41 ± 0.32	22.44 ± 0.67	12.36 ± 0.29
Rugosin E_1_	1.75 ± 0.05	9.18 ± 0.23	13.42 ± 0.39	5.80 ± 0.12
Rugosin E_2_	1.03 ± 0.03	3.28 ± 0.09	5.07 ± 0.14	2.52 ± 0.06
Rugosin D	0.78 ± 0.02	15.62 ± 0.42	19.92 ± 0.52	8.75 ± 0.20
Subtotal	13.09	65.19	91.22	48.07
Other classes				
Protocatechuic acid	0.78 ± 0.02	1.24 ± 0.03	1.32 ± 0.03	0.73 ± 0.01
Gallic acid	11.80 ± 0.34	25.76 ± 0.67	26.17 ± 0.68	19.25 ± 0.40
Ellagic acid	10.92 ± 0.32	26.72 ± 0.77	22.64 ± 0.61	15.27 ± 0.36
Methyl salicylate	0.71 ± 0.02	0.79 ± 0.02	0.97 ± 0.03	0.92 ± 0.02
Caffeic acid	1.08 ± 0.03	3.88 ± 0.12	7.66 ± 0.22	4.20 ± 0.09
1,3-Di-*O*-caffeoylquinic acid	6.18 ± 0.17	11.24 ± 0.34	12.47 ± 0.31	10.43 ± 0.23
Subtotal	31.47	69.63	71.23	50.80
Total	59.07	184.09	214.25	137.62

tr.—traces amounts (<limit of quantification).

**Table 4:** Composition of the essential oils from the flowers of four *Filipendula* species.

Compound	RI	MI ^a^	*F. camschatica*	*F. denudata*	*F. stepposa*	*F. ulmaria*
Simple phenols						
Benzaldehyde	956	i, ii, iii	0.9	3.2	2.9	2.3
Benzyl alcohol	1031	i, ii, iii	0.5	3.0	2.2	1.4
Salicylaldehyde	1043	i, ii, iii	9.0	45.9	25.7	35.7
Ethyl benzoate	1170	i, ii, iii	0.3	0.8	0.9	0.3
Methyl salicylate	1192	i, ii, iii	73.9	20.7	14.2	18.4
Ethyl salicylate	1386	i, ii, iii	5.2	2.4	0.3	1.1
Vanillin	1400	i, ii, iii	tr.	0.3	5.9	0.9
Benzyl salicylate	1872	i, ii, iii	4.4	1.2	0.3	6.3
Subtotal			94.2	77.5	52.4	66.4
Monoterpenes						
Linalool	1098	i, ii, iii	0.2	2.2	5.7	2.3
α-Terpineol	1190	i, ii, iii	0.3	1.1	0.9	2.2
Geraniol	1252	i, ii, iii	0.1	0.5	1.3	0.3
Subtotal			0.6	3.8	7.9	4.8
Sesquiterpenes						
β-Caryophyllene	1420	i, ii, iii	0.4	1.5	1.9	1.6
Humulene	1455	i, ii, iii	0.2	1.3	1.1	0.9
Germacrene D	1483	i, ii, iii	0.2	0.9	1.2	1.2
β-(*E*)-Ionone	1489	i, ii, iii	0.2	1.9	2.7	2.3
δ-Amorphene	1510	i, ii	0.3	0.4	0.3	0.4
Caryophyllene oxide	1587	i, ii	tr.	0.1	tr.	tr.
(*E*)-Asarone	1686	i, ii	0.1	0.2	0.3	0.6
Subtotal			1.4	6.3	7.5	7.0
Aliphatic compounds						
Decanal	1205	i, ii, iii	tr.	0.9	2.9	1.6
Dodecanal	1408	i, ii, iii	tr.	0.2	1.3	0.3
Tetradecanal	1610	i, ii	tr.	0.3	0.9	0.4
Pentadecanal	1712	i, ii	0.1	1.0	2.2	1.9
Hexadecanal	1816	i, ii	2.9	1.3	6.3	5.2
Heptadecanal	1919	i, ii	0.2	5.7	9.4	6.9
*n*-Docosane	2200	i, ii	tr.	0.2	0.9	0.3
*n*-Tricosane	2300	i, ii	tr.	0.1	1.2	0.2
*n*-Tetracosane	2400	i, ii	tr.	0.1	0.3	0.7
*n*-Pentacosane	2500	i, ii	tr.	0.2	0.9	0.3
Subtotal			3.2	10.0	26.3	17.8
Total			99.4	97.6	94.1	96.0
Yield, % ^b^			0.02	0.07	0.11	0.05

^a^ Methods of identification: i—retention index, ii—mass spectrum, iii—co-injection with authentic sample; ^b^ yield, % of dry plant weight; tr.—traces amounts (<0.1%).

**Table 5:** General parameters and monosaccharide compositions of water soluble polysaccharide fractions of four meadowsweet floral tea decoctions.

Parameter	*F. camschatica*	*F. denudata*	*F. stepposa*	*F. ulmaria*
Yield, mg/g	11.75	13.03	17.07	17.24
Total carbohydrate content, mg/g	973.64 ± 30.47	982.50 ± 28.63	989.47 ± 31.20	980.52 ± 30.39
Uronic acid content, mg/g	102.27 ± 3.40	63.18 ± 2.24	79.30 ± 3.01	90.45 ± 3.43
Protein content, mg/g	23.18 ± 0.61	14.37 ± 0.35	12.60 ± 0.30	17.22 ± 0.49
Reaction with I_2_	positive	positive	positive	positive
Reaction with resorcinol	negative	negative	negative	negative
Reaction with Yariv reagent	positive	positive	positive	positive
Monosaccharide composition, mol %				
Ara	10.5	4.2	5.1	6.3
Gal	48.8	43.0	46.4	40.5
Glc	23.4	36.4	29.0	33.6
Fuc	0.2	0.5	0.4	0.9
Man	7.0	6.3	6.9	6.0
Rha	0.8	2.5	3.1	1.6
Xyl	0.4	1.1	1.2	2.7
GalA	8.8	5.9	7.8	8.3

tr.—traces amounts (<limit of quantification).

**Table 6:** Inhibitory effect on amylase (Amy), α-glucosidase (Glu) and AGEs formation (AGE) of four meadowsweet floral tea decoctions and reference compounds ^a^.

Method	*F. camschatica*	*F. denudata*	*F. stepposa*	*F. ulmaria*
Amy ^b^	>100	74.80 ± 2.54 ^ii^	89.67 ± 2.78	85.52 ± 2.90 ^ii^
Glu ^b^	~100	78.53 ± 2.28	71.35 ± 2.06 ^iv^	76.14 ± 2.74 ^iv^
AGE ^c^	10.27 ± 0.41 ^v^	52.11 ± 1.87 ^vi^	61.18 ± 2.20 ^vi^	32.90 ± 1.22 ^v^
	**Spiraeoside**	**Rugosin D**	**Methyl salicylate**	**Acarbose**
Amy ^b^	>100	6.27 ± 0.21 ^i^	>100	10.57 ± 0.34 ^i^
Glu ^b^	>100	4.82 ± 0.16 ^iii^	>100	38.60 ± 1.34 ^iii^
AGE ^d^	<5	69.72 ± 2.37	<5	n.d. ^e^

^a^ Average of three analyses (±SD); ^b^ IC_50_, μg/mL; ^c^ inhibitory activity at 1 mg/mL, %; ^d^ inhibitory activity at 0.5 mg/mL, %; ^e^ n.d.—not determined. All values correspond to mean values ± standard deviation of three replicates. Values with different letters (i–vi) indicate statistically significant differences among groups at *p* < 0.05 by one-way ANOVA.

**Table 7:** Antioxidant activity of four meadowsweet floral tea decoctions and reference compounds ^a^.

Method ^b^	*F. camschatica*	*F. denudata*	*F. stepposa*	*F. ulmaria*
DPPH^•^	23.72 ± 0.59 ^ii^	8.13 ± 0.21 ^ii^	7.33 ± 0.19 ^ii^	10.43 ± 0.26 ^ii^
ABTS^•+^	9.76 ± 0.23 ^iv^	4.97 ± 0.11 ^iii^	4.08 ± 0.08 ^iii^	5.74 ± 0.12 ^iii^
Br^•−^	111.89 ± 2.12 ^v^	241.95 ± 4.35 ^vi^	259.64 ± 4.67 ^vi^	228.11 ± 4.33 ^v, vi^
CBA	21.16 ± 0.76	4.11 ± 0.15 ^ix^	3.53 ± 0.12 ^viii, ix^	4.55 ± 0.16 ^ix^
	**Spiraeoside**	**Rugosin D**	**Methyl salicylate**	**Trolox**
DPPH^•^	82.40 ± 2.14	5.36 ± 0.11 ^i^	>100	7.40 ± 0.14 ^i^
ABTS^•+^	7.02 ± 0.14 ^iii, iv^	0.70 ± 0.01	59.18 ± 1.24	4.27 ± 0.08 ^iii^
Br^•−^	1046.82 ± 19.88 ^vii^	1293.63 ± 24.58 ^vii^	331.53 ± 5.96 ^vi^	663.25 ± 12.98
CBA	>100	2.62 ± 0.09	>100	2.70 ± 0.10 ^viii^

^a^ Average of three analyses (±SD); ^b^ DPPH^•^—DPPH^•^ radical scavenging activity (IC_50_, μg/mL); ABTS^•+^—ABTS^•+^ radical scavenging activity (IC_50_, μg/mL); Br^•−^—bromine anion radical scavenging activity (mg gallic acid equivalents per g extract); CBA—carotene bleaching assay (IC_50_, μg/mL). All values correspond to mean values ± standard deviation of three replicates. Values with different letters (i–ix) indicate statistically significant differences among groups at *p* < 0.05 by one-way ANOVA.

## References

[1] Zakaria N.Z.I., Masnan M.J., Zakaria A., Shakaff A.Y.M. (2014). A bio-inspired herbal tea flavor assessment technique. Sensors.

[2] Mukherjee P.K. (2015). Value chains of herbal medicines—Ethnopharmacological and analytical challenges in a globalizing world. Evidence-Based Validation of Herbal Medicine.

[3] Junior E.L.C., Morand C. (2016). Interest of mate (*Ilex paraguariensis* A. St.-Hil.) as a new natural functional food to preserve human cardiovascular health—A review. J. Funct. Foods.

[4] Zengin G., Uysal A., Gunes E., Aktumsek A. (2014). Survey of phytochemical composition and biological effects of three extracts from a wild plant (*Cotoneaster nummularia* Fisch. et Mey.): A potential source for functional food ingredients and drug formulations. PLoS ONE.

[5] Olennikov D.N., Kashchenko N.I., Chirikova N.K., Tankhaeva L.M. (2015). Iridoids and flavonoids of four Siberian gentians: Chemical profile and gastric stimulatory effect. Molecules.

[6] Olennikov D.N., Kashchenko N.I., Chirikova N.K., Koryakina L.P., Vladimirov L.N. (2015). Bitter gentian teas: Nutritional and phytochemical profiles, polysaccharide characterisation and bioactivity. Molecules.

[7] Ghedira K., Goetz P., Jeune R. (2011). Reine-des-prés (sommité fleurie de) *Filipendula ulmariae* (L.) Maxim. Phytothérapie.

[8] Lindeman A., Jounelaeriksson P., Lounasmaa M. (1982). The aroma composition of the flower of meadowsweet (*Filipendula ulmaria* (L.) Maxim). Lebensm. Wiss. Technol..

[9] Toiu A., Vlase L., Oniga I., Benedec D., Tămaş M. (2011). HPLC analysis of salicylic derivatives from natural products. Farmacia.

[10] Blazics B., Papp I., Kery A. (2010). LC-MS qualitative analysis and simultaneous determination of six *Filipendula* salicylates with two standards. Chromatographia.

[11] Wilkes S., Glasl H. (2001). Isolation, characterization, and systematic significance of 2-pyrone-4,6-dicarboxylic acid in *Rosaceae*. Phytochemistry.

[12] Bijttebier S., Van der A.A., Voorspoels S., Noten B., Hermans N., Pieters L., Apers S. (2016). A first step in the quest for the active constituents in *Filipendula ulmaria* (meadowsweet): Comprehensive phytochemical identification by liquid chromatography coupled to quadrupole-orbitrap mass spectrometry. Planta Med..

[13] Bączek K., Cygan M., Przybył J.L., Kosakowska O., Węglarz Z. (2012). Seasonal variation of phenolics content in above- and underground organs of dropwort (*Filipendula vulgaris* Moench). Herba Pol..

[14] Pemp E., Reznicek G., Krenn L. (2007). Fast quantification of flavonoids in *Filipendulae ulmariae* flos by HPLC/ESI-MS using a nonporous stationary phase. J. Anal. Chem..

[15] Abe I., Kashiwagi Y., Noguchi H., Tanaka T., Ikeshiro Y., Kashiwada Y. (2001). Ellagitannins and hexahydroxydiphenoyl esters as inhibitors of vertebrate squalene epoxidase. J. Nat. Prod..

[16] Piwowarski J.P., Granica S., Zwierzyńska M., Stefańska J., Schopohl P., Melzig M.F., Kiss A.K. (2014). Role of human gut microbiota metabolism in the anti-inflammatory effect of traditionally used ellagitannin-rich plant materials. J. Ethnopharmacol..

[17] Radulović N., Mišić M., Aleksić J., Ðoković D., Palić R., Stojanović G. (2007). Antimicrobial synergism and antagonism of salicylaldehyde in *Filipendula vulgaris* essential oil. Fitoterapia.

[18] Pavlovic M., Petrovic S., Ristic M., Maksimovic Z., Kovacevic N. (2007). Essential oil of *Filipendula hexapetala*. Chem. Nat. Comp..

[19] Karimova O.A., Zhigunov O.Y. (2016). Introduction of some varieties of *Filipendula* Mill. genus in Ufa Botanic Garden. Biol. Sci..

[20] Gudkova N.Y. (2012). Perspectives of introduction of meadowsweet (*Filipendula* Mill.) as a source of medicinal raw material. Agric. Biol..

[21] Olennikov D.N., Kruglova M.Y. (2013). A new quercetin glycoside and other phenolic compounds from the genus *Filipendula*. Chem. Nat. Comp..

[22] Aseeva T.A. (2008). Tibetan Medicine of Buryats.

[23] Makarov A.A. (1974). Plant remedies of the Traditional Yakutian Medicine.

[24] (2004). Buryats.

[25] Zykova I.D., Efremov A.A., Gerasimov V.S., Leshok A.A. (2013). Features of the accumulation of macro- andmicronutrients in the aboveground parts of *Filipendula ulmaria* (L.) Maxim. in different phonological phases. Chem. Plant Raw Mater..

[26] Enel M. (2003). Distribution of heavy metals in plants and their habitats in the outcrop area of Dictyonema shale. Oil Shale.

[27] Szefer P., Nriagu J.O. (2007). Mineral Components in Foods.

[28] Valle M.G., Nano G.M., Tira S. (1988). The essential oil of *Filipendula ulmaria*. Planta Med..

[29] Wu Y., Meng X., Liu X., Bao Y., Wang S. (2005). Analysis of essential oils from flower-buds, leaves and stems of *Filipendula palmata* (Pall.) Maxim. Chem. Res. Chin. Univ..

[30] Kačuráková M., Capek P., Sasinková V., Wellner N., Ebringerová A. (2000). FT-IR study of plant cell wall model compounds: Pectic polysaccharides and hemicelluloses. Carbohydr. Polym..

[31] Arifkhodzhaev A.O. (2000). Galactans and galactan-containing polysaccharides of higher plants. Chem. Nat. Comp..

[32] De Sales P.M., de Souza P.M., Simeoni L.A., de Olivera Magalhães P., Silveira D. (2012). α-Amylase inhibitors: A review of raw material and isolated compounds from planr source. J. Pharm. Pharm. Sci..

[33] Sugimoto K., Yasujima M., Yagihashi S. (2008). Role of advanced glycation end products in diabetic neuropathy. Curr. Pharm. Des..

[34] Etxeberria U., de la Garza A.L., Campión J., Martínez J.A., Milagro F.I. (2012). Antidiabetic effects of natural plant extracts via inhibition of carbohydrate hydrolysis enzymes with emphasis on pancreatic alpha amylase. Expert Opin. Ther. Targets.

[35] Li H., Tanaka T., Zhang Y.J., Yang C., Kouno I. (2007). Rubusuaviins A-F, monomeric and oligomeric ellagitannins from Chinese sweet tea and their alpha-amylase inhibitory activity. Chem. Pharm. Bull..

[36] Xiao J., Ni X., Kai G., Chen X. (2013). A review on structure-activity relationships of dietary polyphenols inhibiting α-amylase. Crit. Rev. Food Sci. Nutr..

[37] Yin Z., Zhang W., Feng F., Zhang Y., Kang W. (2014). α-Glycosidase inhibitors isolated from medicinal plants. Food Sci. Hum. Wellness.

[38] Peng X., Ma J., Chen F., Wang M. (2011). Naturally occurring inhibitors against the formation of advanced glycation end-products. Food Funct..

[39] Selenge E., Odontuya G., Murata T., Sasaki K., Kobayashi K. (2013). Phytochemical constituents of Mongolian traditional medicinal plants, *Chamaerhodos erecta* and *C. altaica*, and its constituents prevents the extracellular matrix degradation factors. J. Nat. Med..

[40] Pukalskienė M., Venskutonis P.R., Pukalskas A. (2015). Phytochemical characterization of *Filipendula ulmaria* by UPLC/Q-TOF-MS and evaluation of antioxidant activity. Rec. Nat. Prod..

[41] Katanić J., Boroja T., Stanković N., Mihailović V., Mladenović M. (2015). Bioactivity, stability and phenolic characterization of *Filipendula ulmaria* (L.) Maxim. Food Funct..

[42] Maksimović Z., Petrović S., Pavlović M., Kovačević N., Kukić J. (2007). Antioxidant activity of *Filipendula hexapetala* flowers. Fitoterapia.

[43] Katanić J., Mihailović V., Stanković N., Boroja T., Mladenović M. (2015). Dropwort (*Filipendula hexapetala* Gilib.): Potential role as antioxidant and antimicrobial agent. EXCLI J..

[44] Pukalskiene M., Venskutonis P.R., Pukalaskas A. (2015). Phytochemical composition and antioxidant properties of *Filipendula vulgaris* as a source of healthy functional ingredients. J. Funct. Foods..

[45] Samuelsen A.B., Lund I., Djahromi J.M., Paulsen B.S., Wold J.K. (1999). Structural features and anti-complementary activity of some heteroxylan polysaccharide fractions from the seeds of *Plantago major* L.. Carbohydr. Polym..

[46] Kiyohara H., Yamada H. (1989). Structure of an anti-complementary arabinogalactan from the root of *Angelica acutiloba* Kitagawa. Carbohydr. Res..

[47] Samuelsen A.B., Paulsen B.S., Wold J.K., Knutsen S.H., Yamada H. (1998). Characterization of a biologically active arabinogalactan from the leaves of *Plantago major* L.. Carbohydr. Polym..

[48] Varljen J., Lipták A., Wagner H. (1989). Structural analysis of a rhamnoarabinogalactan and arabinogalactans with immuno-stimulating activity from *Calendula officinalis*. Phytochemistry.

[49] (2013). Organoleptic Analysis of Herbal Ingredients.

[50] (2011). Quality Control Methods for Herbal Materials.

[51] Bradford M.M. (1976). A rapid and sensitive method for the quantification of microgram quantities of protein utilizing the principle of protein-dye binding. Anal. Biochem..

[52] Dubois M., Gilles K.A., Hamilton J.K., Rebers P.A., Smith F. (1956). Colorimetric method for determination of sugars and related substances. Anal. Chem..

[53] Scholtze H. (1985). Determination of phenylthiocarbamyl amino acids by reversed-phase high-performance liquide chromatography. J. Chromatogr..

[54] Wolf R.E., Adams M. (2015). Multi-Elemental Analysis of Aqueous Geochemical Samples by Quadrupole Inductively Coupled Plasma-Mass Spectrometry (ICP-MS).

[55] Sevag M.G., Lackman D.B., Smolens J. (1938). The isolation of the components of *Streptococcal nucleoproteins* in serologically active form. J. Biol. Chem..

[56] Olennikov D.N., Tankhaeva L.M., Samuelsen A.B. (2006). Quantitative analysis of polysaccharides from *Plantago major* using the Dreywood method. Chem. Nat. Comp..

[57] Usov A.T., Bilan M.I., Klochkova N.G. (1995). Polysaccharides of algae. 48. Polysaccharide composition of several calcareous red algae: Isolation of alginate from *Corallina*
*pilulitara* P. et R. (Rhodophyta, Corallinaceae). Bot. Mar..

[58] Olennikov D.N., Stolbikova A.V., Rokhin A.V., Khobrakova V.B., Tankhaeva L.M. (2011). Polysaccharides from Fabaceae. V. α-Glucan from *Sophora flavescens* roots. Chem. Nat. Comp..

[59] Olennikov D.N., Tankhaeva L.M. (2011). A quantitative assay for total fructans in burdock (*Arctium* spp.) roots. Russ. J. Bioorg. Chem..

[60] Togola A., Inngjerdingen M., Diallo D., Barsett H., Rolstad B. (2007). Polysaccharides with complement fixing and macrophage stimulation activity from *Opilia*
*celtidifolia*, isolation and partial characterization. J. Ethnopharmacol..

[61] Olennikov D.N., Rokhin A.V. (2013). Water-soluble glucans from true cardamom (*Elettaria*
*cardamomum* White at Maton) seeds. Appl. Biochem. Microbiol..

[62] Olennikov D.N., Kashchenko N.I. (2014). Componential profile and amylase inhibiting activity of phenolic compounds from *Calendula officinalis* L. leaves. Sci. World J..

[63] Elya B., Basah K., Mun’im A., Yuliastuti W., Bangun A., Septiana E.K. (2012). Screening of α-glucosidase inhibitory activity from some plants of Apocynaceae, Clusiaceae, Euphorbiaceae, and Rubiaceae. BioMed Res. Int..

[64] Matsuura N., Aradate T., Sasaki C., Kojima H., Ohara M. (2002). Screening sysytem for the Maillard reaction inhibitor from natural product extract. J. Health Sci..

[65] Olennikov D.N., Kashchenko N.I., Chirikova N.K. (2014). A novel HPLC-assisted method for investigation of the Fe^2+^-chelating activity of flavonoids and plant extracts. Molecules.

[66] Asker M.M.S., Shawky B.T. (2010). Structural characterization and antioxidant activity of an extracellular polysaccharide isolated from *Brevibacterium otitidis* BTS 44. Food Chem..

[67] Ding H., Chou T., Liang C. (2010). Antioxidant and antimelanogenic properties of rosmarinic acid methyl ester from *Origanum vulgare*. Food Chem..

[68] Ziyatdinova G., Salikhova I., Budnikov H. (2014). Coulometric titration with electrogenerated oxidants as a tool for evaluation of cognac and brandy antioxidant properties. Food Chem..

[69] Olennikov D.N., Tankhaeva L.M., Agafonova S.V. (2011). Antioxidant components of *Laetiporus sulphureus* (Bull.: Fr.) Murr. fruit bodies. Appl. Biochem. Microbiol..

[70] Michaelsen T.E., Gilje A., Samuelsen A.B., Hogasen K., Paulsen B.S. (2000). Interaction between human complement and a pectin type polysaccharide fraction, PMII, from the leaves of *Plantago major* L.. Scand. J. Immunol..

[71] Olennikov D.N., Tankhaeva L.M. (2007). Lamiaceae carbohydrates. 1. Pectinic substances and hemicelluloses from *Mentha*
*× piperita*. Chem. Nat. Comp..

